# Thermal Transitions in P3HT:PC60BM Films Based on Electrical Resistance Measurements

**DOI:** 10.3390/polym12071458

**Published:** 2020-06-30

**Authors:** Barbara Hajduk, Henryk Bednarski, Marian Domański, Bożena Jarząbek, Barbara Trzebicka

**Affiliations:** Centre of Polymer and Carbon Materials, Polish Academy of Sciences, 34 Marie Curie-Skłodowska str., 41-819 Zabrze, Poland; hbednarski@cmpw-pan.edu.pl (H.B.); mdomanski@cmpw-pan.edu.pl (M.D.); bjarzabek@cmpw-pan.edu.pl (B.J.)

**Keywords:** electrical resistance, spectroscopic ellipsometry, polymer films, organic semiconductors, ellipsometric modeling

## Abstract

In this paper, we present research on thermal transition temperature determination in poly (3-hexylthiophene-2,5-diyl) (P3HT), [6,6]-phenyl-C61-butyric acid methyl ester (PC60BM), and their blends, which are materials that are conventionally used in organic optoelectronics. Here, for the first time the results of electrical resistance measurements are explored to detect thermal transitions temperatures, such as glass transition T_g_ and cold crystallization T_cc_ of the film. To confirm these results, the variable-temperature spectroscopic ellipsometry studies of the same samples were performed. The thermal transitions temperatures obtained with electrical measurements are well suited to phase diagram, constructed on the basis of ellipsometry in our previous work. The data presented here prove that electrical resistance measurements alone are sufficient for qualitative thermal analysis, which lead to the identification of characteristic temperatures in P3HT:PC60BM films. Based on the carried studies, it can be expected that the determination of thermal transition temperatures by means of electrical resistance measurements will also apply to other semi-conducting polymer films.

## 1. Introduction

Organic photovoltaic devices (OPV), such as organic solar cells, are important examples of practical applications of organic optoelectronic materials. For this reason, these materials have been widely studied and described in the literature [1,2,3]. A very significant limitation in the wide application of organic photovoltaic solar cells is their much shorter lifetime when compared to their inorganic counterparts. To achieve further progress, it is also necessary to develop thermal analysis methods [4,5] to examine the thermo-optical properties of thin organic films. The correct determination of these physical properties plays a decisive role in improving their parameters, which, in turn, determines the thermal durability and performance of the final devices. In organic optoelectronics, the most widely studied and used organic materials are semiconducting poly (3-hexylthiophene) (P3HT) [6,7,8,9,10] and its fullerene derivative, [6,6]-phenyl-C_61_-butyric acid methyl ester (PC60BM) [11,12,13,14,15]. Films constructed with blends of both compounds [16,17,18,19,20,21,22] are widely used as active layers in OPV devices [23,24,25]. Solar cells that are based on active layers of P3HT:PC60BM blends commonly serve as reference devices for cells made of other organic materials [26,27,28,29,30]. The maximum reported values of power conversion efficiency of these cells range from 5% to 16–18% [31,32,33]. Cell efficiency is affected by many factors, including surface morphology [34,35] or technical parameters of their preparation process [36,37,38,39,40]. It should be noted that the power conversion efficiency of OPV devices can be optimized by heat treatment. For this purpose, the thermal transition temperatures of OPV materials must be known. Pearson et al. [37] showed that OPV devices were most efficient when the P3HT:PC60BM blends were annealed above the upper apparent glass transition temperature (T_g_) of the blend. They also related optimum values of sample annealing temperature with the content of PC60BM in the studied samples. To confirm the results, two experimental methods were used for T_g_ determination, namely variable-temperature spectroscopic ellipsometry and dynamic mechanical thermal analysis.

Spectroscopic ellipsometry is an experimental optics technique that enables accurate, indirect measurements of thickness and dielectric properties of film materials [41,42,43,44]. A spectroscopic ellipsometer equipped with a temperature adapter enables the thermal analysis of the tested sample by optical measurements during controlled heating or cooling. Literature studies indicate that the characteristic temperatures of thermal transitions can be determined by analysing raw ellipsometric data on the basis of ellipsometric angles or their temperature derivatives [45,46,47,48,49,50,51,52,53]. The temperature dependence of physical quantities, such as film thickness, its coefficient of thermal expansion, refractive index, and ellipsometric data modelling [54,55,56,57,58,59,60] is also explored. In this work we use raw ellipsometric data as the reference results for thermal transition detection, as shown in our previous papers [61,62].

In the literature, there are examples of investigations on thermal transitions of polymers while using measurements of electrical resistance as a function of temperature [63,64,65,66,67]. These literature examples are quite few. Farbod and Tadavani [63] showed that the T_g_ of polyaniline/multiwalled carbon nanotubes composite can be determined by measuring resistance as a function of temperature. Polyaniline and its composites with different content of nanotubes (2, 4, and 16%) were studied. It turned out that the glass transition temperature increased with the concentration of nanotubes. Mei and Chung [65] showed that electrical resistance can be useful for studying thermal properties and thermal history of carbon fiber reinforced nylon-6 composite. They observed the influence of heat treatment on thermal transitions (T_g_ and T_m_) of the materials. It has been confirmed that this measurement method is more sensitive to thermal transitions than the DSC technique (which was the reference method). In this connection, related research carried out on P3HT layers should also be described. Namely, Liu et al. [67] studied P3HT with high molecular weight (Mw) and different regioregularity, while taking their anisotropy into account by analyzing the temperature dependence of conductivity in parallel (σ‖) and perpendicular (σ⊥) directions to the film surface. In the case of highly oriented P3HT thick films, it was found that the temperature relationship σ⊥ reflects the amorphous nature of the charge hopping along this direction. However, σ‖ dropped significantly above 50 °C, which was attributed to a decrease in the in-plane -stacking that was caused by both the melting of crystallized side chains and the enhanced side-chain disturbance with increasing temperature; thus, to the morphological changes within P3HT layer. The abovementioned works used various techniques for measuring resistance, among others, the four-probe technique [63,65], the technique of two electrodes, in which the electrodes were located under or above the foil [64,67].

In this work, for the first time, the possibility of determining the characteristic thermal transition temperatures in nanometric polymer films using temperature-dependent electrical resistance measurements was investigated. For this purpose we used the technique of two-electrodes to measure resistance (the electrodes were placed on the surface of the film) combined with simultaneous ellipsometric measurement. Well-known reference materials (P3HT, PC60BM) were used to demonstrate the usefulness of the method. On the example of films from these materials, it was shown that the thermal transition temperatures can be accurately determined and the results obtained from the electrical resistance are similar to those that were obtained by means of variable temperature spectroscopic ellipsometry and UV-Vis absorption spectroscopy.

We show that electrical resistance measurements that are dependent on temperature, like variable–temperature spectroscopic ellipsometry, are very useful for qualitative thermal analysis, helping to identify characteristic temperatures in films of P3HT:PC60BM blends. These studies are an introduction to the application of this method to other polymer films.

## 2. Materials and Methods

**Figure 1:**
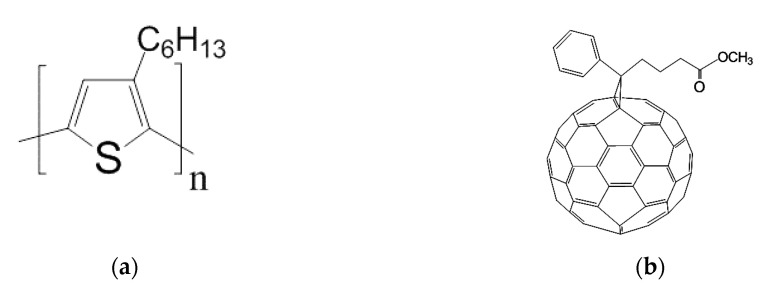
Structures of (**a**) P3HT and (**b**) PC60BM.

The materials used were 95.7% regioregularity poly(3-hexylthiophene-2,5-diyl) M102 (P3HT) (with molar mass Mw = 65.2 kDa) and 99 wt. % purity [6,6]-phenyl-C61-butyric acid methyl ester M112 (PC60BM). All of the materials used were supplied by Ossila. Figure 1 shows their chemical structures.

**Figure 2:**
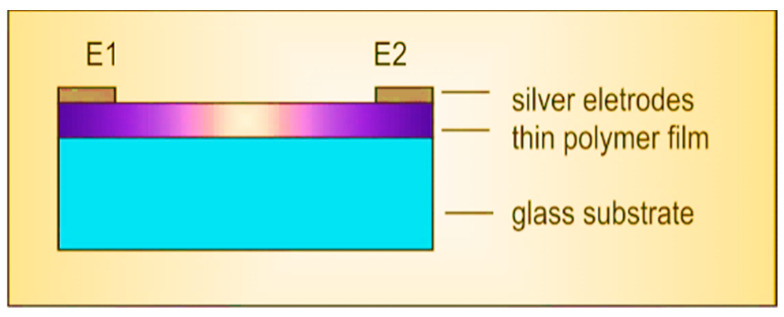
Arrangement of electrodes for measuring electrical resistance.

P3HT, PC60BM, and their 1:1 weight mixture were dissolved in chlorobenzene (maintaining a constant weight concentration of 20 mg/mL) and stirred for 24 h at 60 °C. Films of P3HT, PC60BM, and the P3HT:PC60BM blend were deposited, from these solutions, on microscope glasses (0.1 mm thick) and quartz substrates by spin coating at spin speeds of 750 and 2000 rpm, respectively. The electrodes were applied to the sample surfaces using silver paste. Figure 2 shows the arrangement of the electrodes. All of the samples were stored at room temperature in the laboratory dry box.

Ellipsometric measurements were performed using a SENTECH SE850E spectroscopic ellipsometer (SENTECH Instruments GmbH, Berlin, Germany), operating in the spectral range of 240–2500 nm, while using the Spectra Ray 3 software. Additionally, the ellipsometer is equipped with a variable temperature chamber, operating at low pressure, and an INSTEC mK1000 temperature controller (Instec, Inc., Boulder, CO, USA). The design of the temperature cell allows for measurement at a 70° angle of incidence. The set temperature is precisely maintained by the controller using an electric heater and liquid nitrogen circuit.

The following measurement protocol was used. Each sample was annealed at 200 °C for 2 min and then cooled to 40 °C. The annealing temperature was lower than the thermal degradation temperatures of P3HT and PC60BM [68,69,70,71]. Ellipsometric measurements were carried out during the heating cycle, at a heating rate of 2 °C/min. Measurements were made in the spectral range of 240–930 nm with a time interval of 10 s between subsequent measurements. All of the measurements were carried out in an air atmosphere. Electrical resistance was measured using a Keithley 6517A electrometer/high resistance meter (Keithley Intsruments, Solon, USA) simultaneously with ellipsometric measurements.

Temperature-dependent UV-Vis absorption measurements were carried out using a JASCO V-570 UV-Vis-NIR (200–2500 nm), double-beam spectrophotometer (JASCO Corporation, Tokyo, Japan), controlled by the Spectra Manager program. The prepared films were subjected to gradual annealing in a homemade, electrically-heated sample holder, mounted in the JASCO spectrophotometer, which records transmission spectra at elevated temperatures with an accuracy of ±0.5 °C. The transmission spectra of the films were measured in the temperature range of 20 to 210 °C, with a temperature step of 20 °C.

## 3. Results and Discussion

**Figure 3:**
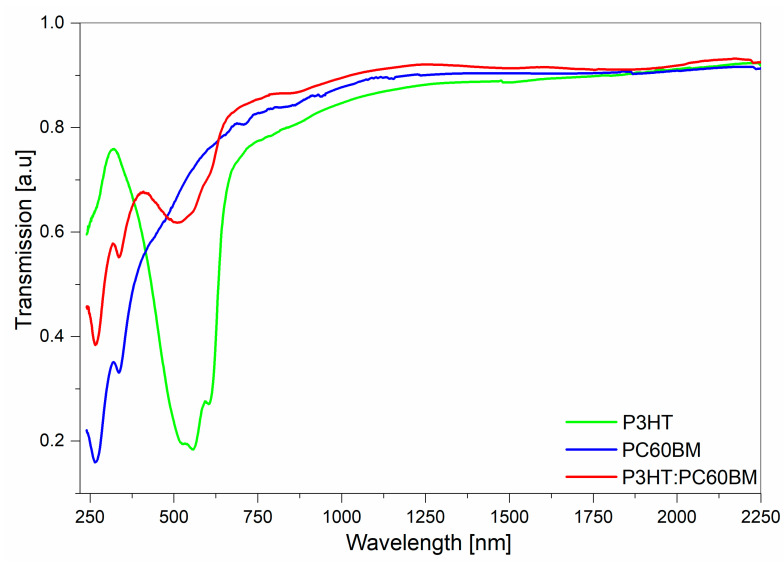
Spectra of P3HT, PC60BM, and P3HT:PC60BM taken in ellipsometer transmission mode.

Figure 3 shows ellipsometric transmission spectra, normalized to unity, for P3HT, PC60BM, and P3HT:PC60BM films deposited on quartz substrates. These spectra were recorded at room temperature in ellipsometer transmission mode. The absorption bands in all of these films are in the spectral range of 240–750 nm. This indicates that the Cauchy dispersion model can be applied to the samples, for wavelengths above 750 nm, in order to determine film thickness.

The Cauchy model parameterizes the spectral dispersion of the refractive index, *n*(*λ*)*,* and the extinction coefficient, *k*(*λ*), as follows [61,62]:

(1) $$n\left(\lambda \right)=n_{0}+C_{0}\frac{n_{1}}{\lambda^{2}}+C_{1}\frac{n_{2}}{\lambda^{4}}$$

and

(2) $$k\left(\lambda \right)=k_{0}+C_{0}\frac{k_{1}}{\lambda^{2}}+C_{1}\frac{k_{2\;}}{\lambda^{4}}$$

where the temperature-dependent parameters *n_i_* and *k_i_* (*i* = 0, 1, 2) are the model parameters, and the coefficients *C*_0_ and *C*_1_ are numerical constants. Table 1 shows the thicknesses of the studied samples determined using this model.

**Figure 4:**
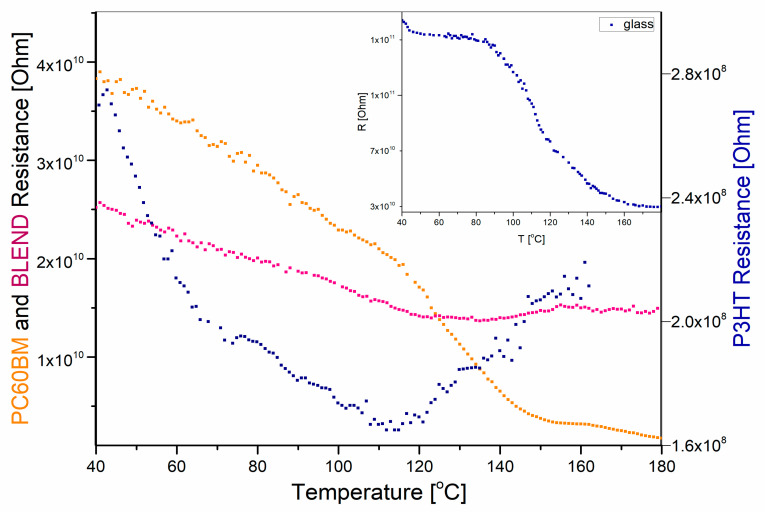
Electrical resistance as a function of temperature of indicated P3HT, PCBM and P3HT:PCBM blend films deposited on a glass substrates. Inset: glass resistance.

Figure 4 shows the measured resistances of the spin coated films, measured on glass substrates, as a function of temperature.

The electrical resistance of the glass substrate alone is one to three orders of magnitude higher than that measured for coated films on the substrates, as can be seen from Figure 4. Nevertheless, an equivalent electrical circuit can be adopted as a parallel connection of resistors representing, respectively, film and substrate in order to take into account the corresponding leakage current. Therefore, for electrical conductance of the film-substrate, $\sigma_{film/substrate}=1/R$, the following relation applies:

(3) $$\sigma_{film/substrate}=\sigma_{film}+\sigma_{substrate}$$

where the meaning of the subscripts for conductance are self-explanatory. In practice, the resistance of the glass substrate and the resistance of the film-substrate system are measured, and the change in the value of the electrical resistance of the film itself should be calculated while using the above formula. It should be noted that the conductivity value determined by us for P3HT film, which is of the order 10^−5^ S/cm, agrees well with the value determined by Liu et al. [67] for a 15× thicker P3HT layer.

**Figure 5:**
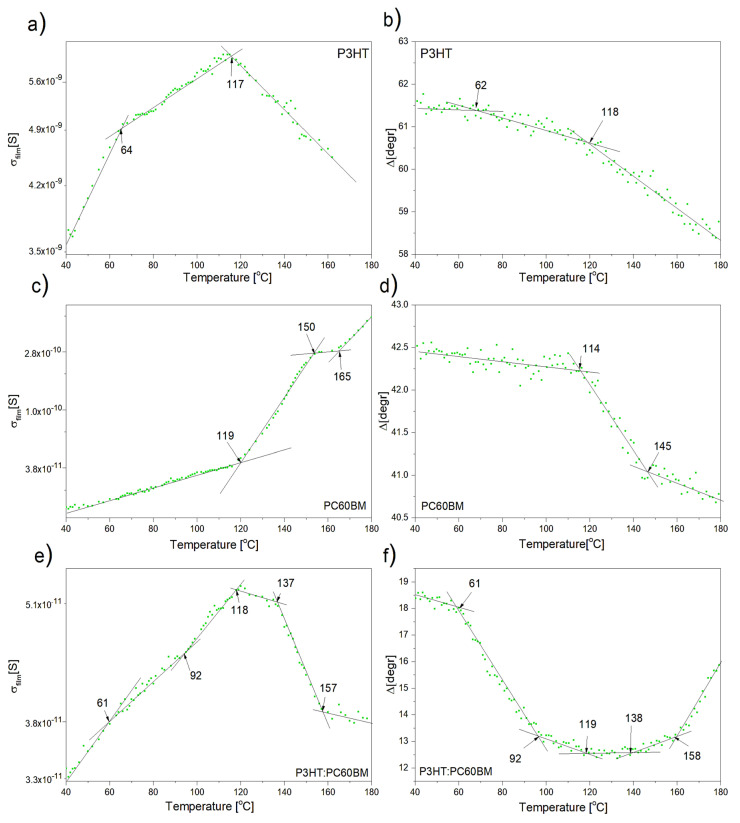
Left panel: The electrical conductance as a function of temperature for: (**a**) P3HT, (**c**) PC60BM, and (**e**) P3HT:PC60BM (1:1) blend. Right panel: The ellipsometric angle Δ at 280 nm as a function of temperature for: (**b**) P3HT, (**d**) PC60BM, and (**f**) P3HT:PC60BM blend.

The temperature dependence of the electrical conductance, $\sigma_{film}$*(T)* and ellipsometric angle *Δ(T)* obtained for films of P3HT, PC60BM, and their 1:1 weight blend is shown in Figure 5, on the left and right panel, respectively. It should be noted that these relationships are presented on a logarithmic scale, which reveals a qualitative similarity to the corresponding *Δ(T)* ellipsometric curves that are shown on the right panel. This similarity indicates the possibility of performing a linear analysis, also, for $\sigma_{film}$*(T)* to determine the characteristic temperatures of the thermal transitions. Linear analysis involves fitting straight lines using the least squares method. Fittings were made in subsequent recorded sections of ellipsometric and resistance curves. Let’s take a closer look on results for pure materials. From the *Δ(T)* relationship for P3HT, two characteristic temperatures of 64 and 118 °C can be distinguished, while the corresponding characteristic temperatures on the conductance curve are 62 and 117 °C. This is a one-to-one correspondence. Based on our previous work [61,62], each of them can be identified. The lower transition temperatures, i.e., 62 °C on *Δ(T)* and 64 °C on $\sigma_{film}$*(T)*, correspond to the cold crystallization temperatures T_cc_ of P3HT. In the case of the PC60BM film, the temperature dependence of the electrical conductance curve shows three characteristic temperatures: 119, 150, and 165 °C. However, only two characteristic temperatures are clearly visible on the curve *Δ(T)*, at 116 and 145 °C. This is most likely due to the large dispersion of ellipsometric data at temperatures above 140 °C. Comparing the charts for *Δ(T)* and $\sigma_{film}$*(T)* of the pure materials, several important conclusions can be drawn. Namely, with increasing temperature, the conductance of P3HT increases until reaching second cold crystallization temperature T_cc2_ = 117 °C. When this temperature is exceeded, the trend reverses. Similarly, the conductance of the blend increases until reaching 118 °C. However, the conductance of PC60BM grows in the whole range. After exceeding T_g_, the growth rate of $\sigma_{film}$*(T)* for PC60BM increases significantly.

**Figure 6:**
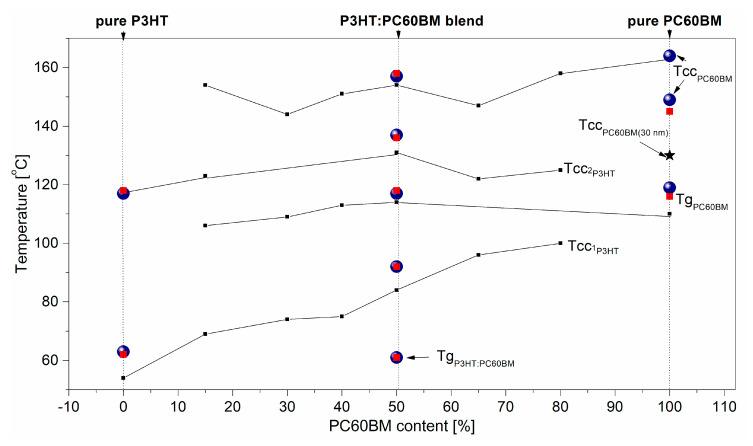
The characteristic temperatures of thermal transitions in P3HT:PC60BM (1:1) films, obtained from resistance and ellipsometric measurements (blue balls and red squares, respectively) added to the phase diagram presented earlier in [61]. The point marked with an asterisk represents the T_cc_ temperature of 30 nm thick PC60BM film.

For films of P3HT:PC60BM blends, corresponding charts for *Δ(T)* and $\sigma_{film}$*(T)* contain a larger number of characteristic temperatures. Zhao et al. [72] showed that, even in a single-phase of P3HT: PCBM blends several characteristic temperatures originated from various thermal transitions can be observed in DSC measurements. In the case of a multiphase system (whose presence in our case has been confirmed by means of an optical microscope and UV-Vis absorption spectroscopy), additional thermal transitions and, consequently, characteristic temperatures from the precipitated phases P3HT and PC60BM can be expected. That is why we used the phase diagram published in [61] to identify thermal transition temperatures for the film of P3HT:PCBM blend, see Figure 6. In this way, temperatures of 118 °C, 137, and 157 on the plot for $\sigma_{film}$*(T)* of the blend can be identified as glass transition temperature of PC60BM and cold crystallization temperatures of the P3HT and PC60BM phases, respectively. However, the characteristic temperature at about 92 °C in the blend should be identified as T_cc1_ of P3HT phase. As can be seen in Figure 6, the values of the characteristic temperatures determined on the basis of resistance measurements in this work, blue balls, are similar to the corresponding characteristic temperatures that were previously determined on the basis of only ellipsometric results.

**Figure 7:**
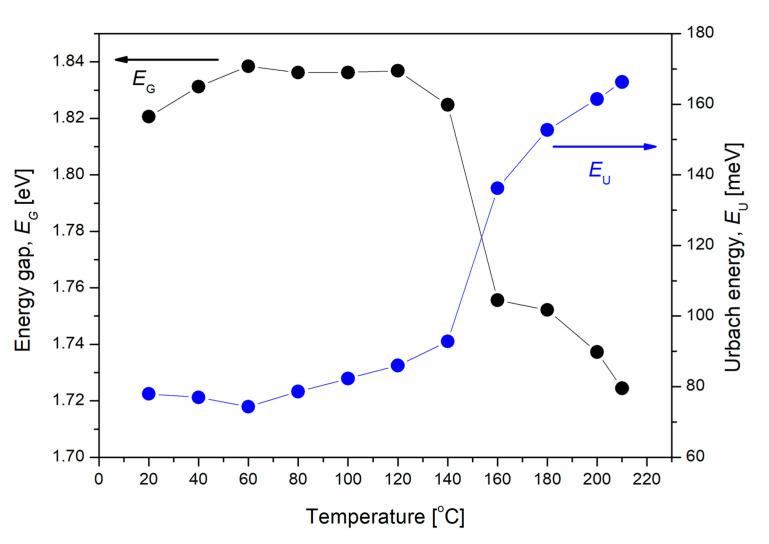
The absorption edge parameters: the energy gap (*E_G_*) and the Urbach energy (*E_U_*) as a function of temperature for P3HT:PC60BM film deposited on quartz substrate.

The identification of the thermal transition at 157 °C was also confirmed by temperature-dependent UV-Vis absorption measurements. It is related to the ordering and formation of defects (precipitation) of the PC60BM phase in P3HT:PC60BM films. Figure 7 shows the temperature dependence of the absorption edge parameters, energy gap (*E_G_*), and Urbach energy (*E_U_*), which are associated with the length of conjugation and structural disorder, respectively.

The values of the absorption edge in Figure 7 were determined on the basis of absorption measurements in situ, during annealing of the P3HT:PC60BM blend film, up to 210 °C. The curves *E_G_(T)* and *E_U_(T)* reflect increasing processes of P3HT order, the orderly stacking of polymer chains, and the introduction of structural defects within the flexible side chains, which are responsible for slight changes of the absorption edge parameters. After exceeding the glass transition temperature of the PC60BM film, the increase in *E_U_* and reduction of *E_G_* can be clearly seen. At temperatures above 140 °C, rapid changes in these parameters can be attributed to the creation of PC60BM clusters that occur with the phase separation of the P3HT:PC60BM mixture. Different behaviors have been observed for the pure PC60BM and P3HT films during their stepwise annealing, up to 200 °C, as presented in [73]. Both the absorption spectra and absorption edge parameters (*E_G_* ≅ 2.73 eV, *E_U_* ≅ 480 meV) of the PC60BM film did not change during thermal treatment. In the case of the P3HT film, the conjugation of polymer chains was not influenced by heating (*E_G_* *≅* 1.86 eV was constant during the annealing/cooling process), while the linear temperature dependence of the *E_U_* was connected with reversible, thermally-induced movements of elastic hexyl side chains.

**Figure 8:**
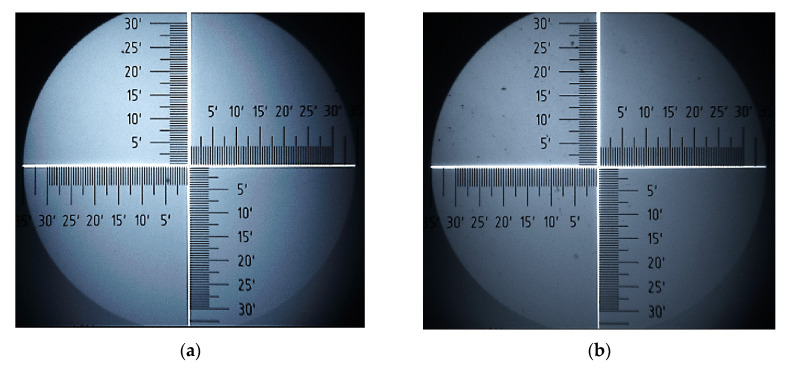
P3HT before (**a**) and after annealing (**b**).

**Figure 9:**
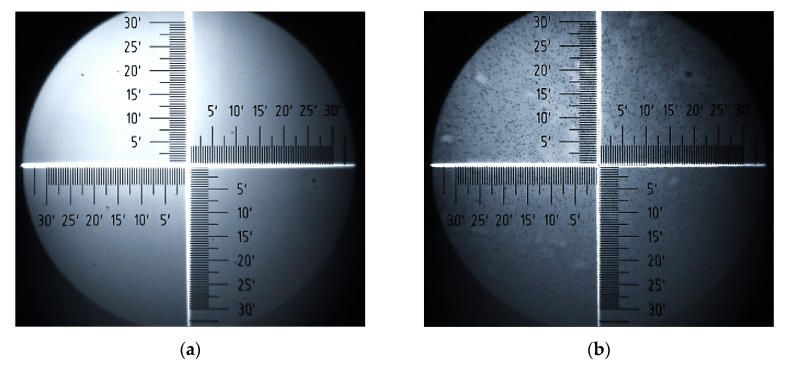
PC60BM before (**a**) and after annealing (**b**).

**Figure 10:**
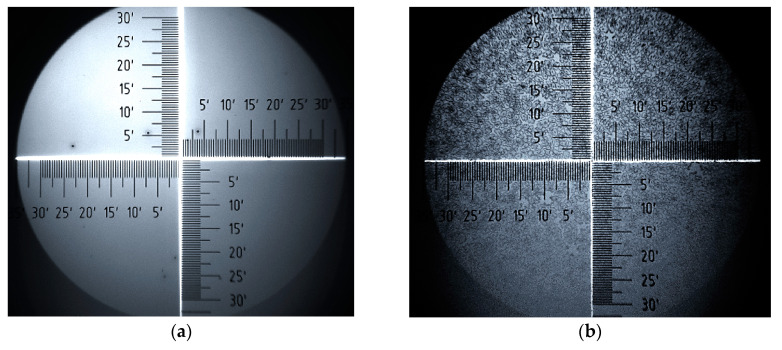
P3HT:PC60BM before (**a**) and after annealing (**b**).

Analysis of surface images of samples, obtained with an optical microscope, provides valuable additional information on changes in surface morphology of P3HT and PC60BM. Figure 8 and Figure 9 show a series of surface images taken before and after thermal annealing, allowing for the comparison of the film surfaces of pure P3HT and PC60BM materials, respectively. Further, Figure 10 shows corresponding images for the P3HT:PC60BM blend film. On the surfaces of all the samples, precipitation appears after annealing. In the case of P3HT (Figure 8b), there are quite a few numerous and widely-spread precipitations on the surface, while the surface of pure PC60BM (Figure 9b) is densely covered with large precipitates. The most ordered phase is formed on the surface of the P3HT:PC60BM (Figure 10b) blend film. After temperature treatment, the entire surface of the sample is covered with interconnected precipitation. Such an intense change is associated with the formation of crystallites of the pure PC60BM phase, included in the P3HT:PC60BM blend film in the temperature range of 130–160 °C.

## 4. Conclusions

The paper presents the results of research on the thermal behavior of P3HT, PC60BM films, and their blends. The measurements of electrical resistance as a function of sample temperature allowed determination of thermal transitions of studied films. This gave us the basis to perform a linear analysis for conductance $\sigma_{film}$*(T)* and allowed reading the characteristic temperatures of thermal transitions directly from the $\sigma_{film}$*(T)* plots. The results of electrical resistance were compared with data that were obtained by other methods (variable-temperature spectroscopic ellipsometry and UV-Vis absorption spectroscopy). As it turned out, temperatures determined by different methods overlap. The results of the measurements of *E_G_(T)* and *E_U_(T)* parameters obtained by means of UV-Vis absorption spectroscopy confirm that the obtained P3HT: PC60BM mixtures are multiphase systems. In addition, the attached microscopic image of the surface of the samples taken before and after temperature measurements shows the occurrence of precipitation. This shows that electrical resistance measurements are very useful for qualitative thermal analysis, helping to identify characteristic temperatures in polymer/fullerene films. Moreover, the thermal transitions temperatures obtained with electrical measurements can be included to phase diagram, constructed on the basis of ellipsometry in our previous work. The presence of thermal transitions, such as glass transitions or cold crystallization, can be detected while using both raw ellipsometric data and electrical resistance measurements. However, it should be remembered that, unlike optical measurements, electrical measurements require contacts on the sample surface, i.e., interference with the sample structure. The work was carried out on the basis of films from materials whose thermal properties are well known and widely described in the literature. However, it can be expected that the presented method can be applied to films that are made of other polymeric materials.

## Figures and Tables

**Table 1:** Sample thicknesses determined by spectroscopic ellipsometry.

Sample		P3HT	PC60BM	P3HT:PC60BM
thickness [nm]	quartz	150	80	120
	microscope glass	250	400	250
